# Massive Gene Expansion and Sequence Diversification Is Associated with Diverse Tissue Distribution, Regulation and Antimicrobial Properties of Anti-Lipopolysaccharide Factors in Shrimp

**DOI:** 10.3390/md16100381

**Published:** 2018-10-11

**Authors:** Gabriel Machado Matos, Paulina Schmitt, Cairé Barreto, Natanael Dantas Farias, Guilherme Toledo-Silva, Fanny Guzmán, Delphine Destoumieux-Garzón, Luciane Maria Perazzolo, Rafael Diego Rosa

**Affiliations:** 1 Laboratory of Immunology Applied to Aquaculture, Department of Cell Biology, Embryology and Genetics, Federal University of Santa Catarina, Florianópolis SC 88040-900, Brazil; gabrielmatos92@gmail.com (G.M.M.); cairebarreto@gmail.com (C.B.); natan.cbio@gmail.com (N.D.F.); l.m.perazzolo@ufsc.br (L.M.P.); 2 Laboratorio de Genética e Inmunología Molecular, Instituto de Biología, Facultad de Ciencias, Pontificia Universidad Católica de Valparaíso, Valparaíso 2373223, Chile; paulina.schmitt@pucv.cl; 3 Department of Cell Biology, Embryology and Genetics, Federal University of Santa Catarina, Florianópolis SC 88040-900, Brazil; guilherme.toledo@ufsc.br; 4 Núcleo Biotecnología Curauma, Pontificia Universidad Católica de Valparaíso, Valparaíso 2373223, Chile; fanny.guzman@pucv.cl; 5 Interactions Hôtes-Pathogènes-Environnements, Université de Montpellier, CNRS, Ifremer, Université de Perpignan Via Domitia, CEDEX 5, 34090 Montpellier, France; Delphine.Destoumieux.Garzon@ifremer.fr

**Keywords:** host defense peptide, antimicrobial peptide, anti-LPS factor, host‒microbe relationship, functional diversity, invertebrate immunity, crustacean, antimicrobial activity

## Abstract

Anti-lipopolysaccharide factors (ALFs) are antimicrobial peptides with a central β-hairpin structure able to bind to microbial components. Mining sequence databases for ALFs allowed us to show the remarkable diversity of ALF sequences in shrimp. We found at least seven members of the ALF family (Groups A to G), including two novel Groups (F and G), all of which are encoded by different loci with conserved gene organization. Phylogenetic analyses revealed that gene expansion and subsequent diversification of the ALF family occurred in crustaceans before shrimp speciation occurred. The transcriptional profile of ALFs was compared in terms of tissue distribution, response to two pathogens and during shrimp development in *Litopenaeus vannamei*, the most cultivated species. ALFs were found to be constitutively expressed in hemocytes and to respond differently to tissue damage. While synthetic β-hairpins of Groups E and G displayed both antibacterial and antifungal activities, no activity was recorded for Group F β-hairpins. Altogether, our results showed that ALFs form a family of shrimp AMPs that has been the subject of intense diversification. The different genes differ in terms of tissue expression, regulation and function. These data strongly suggest that multiple selection pressures have led to functional diversification of ALFs in shrimp.

## 1. Introduction

Anti-lipopolysaccharide factors (ALFs) are multifunctional antimicrobial host defense peptides (AMPs) with the ability to bind to microbial surface molecules. They were initially characterized as potent inhibitors of lipopolysaccharide (LPS)-induced clotting in marine chelicerates, the horseshoe crabs *Tachypleus tridentatus* and *Limulus polyphemus* [1]. In addition to their LPS-binding properties, they were also shown to be highly active against Gram-negative bacteria [2]. In the early 2000s, ALF homologues were identified in hemocyte transcriptomes from two penaeid shrimp, *Litopenaeus setiferus* and *Penaeus monodon* [3,4]. Although ALF sequences have been extensively identified in many species, these AMPs appear to be exclusive of marine chelicerates and crustaceans.

ALFs are genetically encoded as precursor molecules composed of a leader sequence followed by a mature peptide containing two conserved cysteine residues [5]. The three-dimensional structure of both horseshoe crab and shrimp ALFs consists of three α-helices packed against a four-stranded β-sheet [6]. In this structure, the two cysteines flank a central β-hairpin of 20 residues stabilized by a single disulfide bond. This central β-hairpin (also known as “LPS-binding domain” or LPS-BD) is the functional domain of ALFs and holds key charged amino acids involved in the recognition and binding of microbial cell wall components, such as LPS from Gram-negative bacteria, lipoteichoic acid from Gram-positive bacteria and β-glucans from fungi [7]. Indeed, the mechanism of action of ALFs is intimately associated with their ability to bind to those microbial moieties. Notably, ALFs are known to be highly active against a broad range of bacteria, fungi and some enveloped viruses [8].

Different from horseshoe crabs, ALFs form a diverse and multigenic family of AMPs in penaeid shrimp. Shrimp ALFs are composed of five members (Groups A to E), which differ in terms of primary structure and biochemical characteristics [5]. While ALFs from Groups A and D possess anionic properties, Groups B and C are exclusively composed of cationic peptides. Interestingly, while cationic ALFs exhibit potent antimicrobial activities against a broad range of bacterial and fungal strains [9], anionic ALFs from Group D have impaired antimicrobial properties [10]. The limited antibacterial activity of Group D ALFs is likely due to the lack of most residues involved in LPS binding of cationic ALFs from Group B [10]. Group E ALFs were only described in the kuruma prawn *Marsupenaeus japonicus* as cationic (*Mj*ALF-E1) and anionic (*Mj*ALF-E2) peptides, with antimicrobial activity restricted to Gram-negative bacteria [11].

At present, little is known about the evolutionary forces that may have shaped the diversification of ALF sequences in shrimp. To address this question and explore the biological implications of such sequence diversity, we combined a series of molecular, phylogenetic, transcriptional and functional analyses. By using an in silico mapping method, we have identified novel ALF members in different penaeid species. Bayesian phylogenetic reconstructions revealed the existence of seven ALF groups in shrimp: the previously described Groups A to E, and the novel Groups F and G evidenced here. Each ALF group is encoded by a different locus in the shrimp genome. Through a quantitative PCR-based approach, we have assessed the expression of the seven ALF genes in terms of tissue distribution and transcriptional response to two pathogens (*Vibrio harveyi* and WSSV), but also during different shrimp development stages (from fertilized eggs to larval and post-larval stages) in the shrimp *L. vannamei*. Finally, we evaluated the antimicrobial activity of synthetic peptides based on the central β-hairpin of the three novel ALFs identified in *L. vannamei* (Groups E to G) and presented evidence that the sequence diversity of shrimp ALFs can be reflected in their biological properties. Altogether, the tissue distribution, regulation and biological functions of ALF genes reveal that various evolutionary pressures have led to functional diversification of the ALF family in penaeid shrimp.

## 2. Results

### 2.1. ALFs from Penaeid Shrimp Comprise a Diverse Family Composed of Seven Members

**Figure 1:**
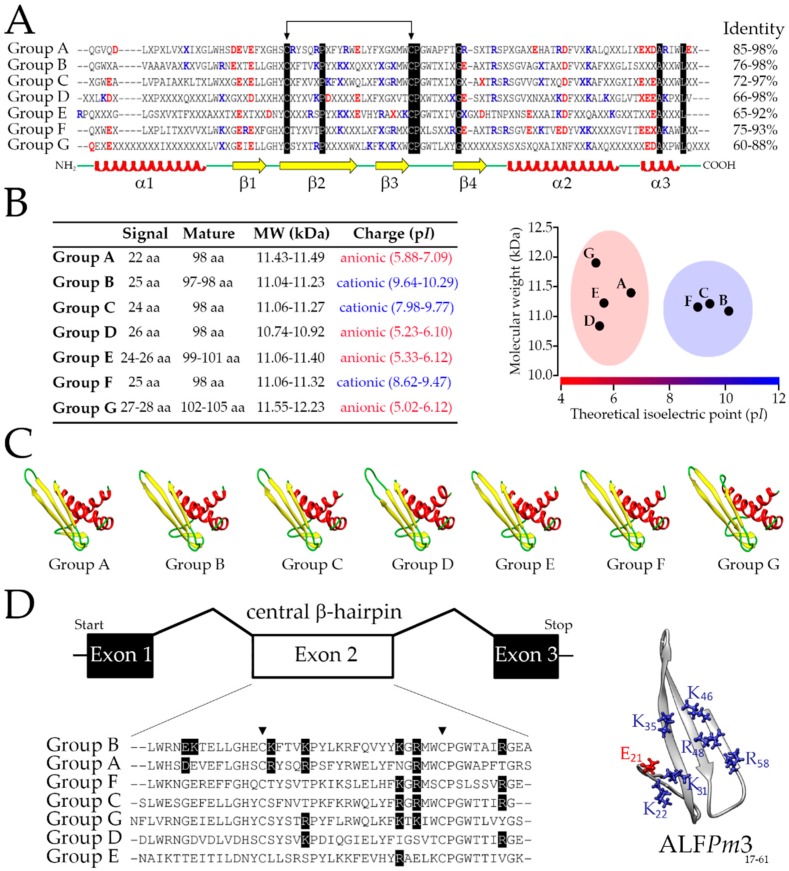
The seven members of the shrimp ALF family. (**A**) Multiple alignments of the consensus amino acid signature of each ALF member (Groups A to G) found in penaeid shrimp. Identical residues are highlighted in black while “X” indicates any amino acid. Positively and negatively charged residues are displayed in blue and red, respectively. The conserved cysteine bond is indicated by the arrows. The position of α-helices (red helices) and β-sheets (yellow arrows) is based on the three-dimensional (3D) structure of ALF*Pm*3 (PDB: 2JOB). Intragroup amino acid identity values are indicated on the right. (**B**) Biochemical properties of shrimp ALFs. MW: molecular weight; p*I*: theoretical isoelectric point; aa: amino acid residues. (**C**) Predicted 3D structure of *L. vannamei* ALFs. The structural models were built based on the NMR (nuclear magnetic resonance) structure of ALF*Pm*3. (**D**) Not-to-scale schematic representation of ALF genes from *P. monodon*: Group A (*ALFPm2*: EF523561), Group B (*ALFPm3*: EF523562), Group C (*ALFPm6*: JN562340), Group D (*ALFPm8*: NIUS010164210), Group E (*ALFPm9*: NIUS010076396), Group F (*ALFPm10*: NIUS011801312) and Group G (*ALFPm11*: NIUS010749450). Boxes represent the exons, and lines represent the introns. Multiple alignments of the amino acid sequences encoded by the exon 2 (white box). Triangles (▼) indicate the two conserved cysteines. Residues involved in LPS binding of ALF*Pm*3 are highlighted in black.

By using an exhaustive in silico screening approach, we recovered 47 unique ALF sequences (complete CDS) from both publicly available annotated and non-annotated databases for 10 penaeid shrimp species (Decapoda: Penaeidae) (Table S1). With all predicted amino acid sequences in hand, we performed multiple alignments in order to classify the obtained sequences into the five previously described ALF groups (Groups A to E). Surprisingly, from our sequence analysis, shrimp ALFs clustered into seven distinct groups with specific amino acid sequence signatures (Figure 1A). In addition to already documented ALFs from Groups A to E, we identified here two novel groups that were conveniently named Group F and G (Figure 1A).

Across shrimp species, ALFs corresponded to full-length transcripts that encode for precursors composed of a signal peptide (22 to 28 residues), followed by a mature peptide (10.74 to 12.23 kDa) containing two conserved cysteine residues (Figure 1A). Besides their differences in size and molecular weight, shrimp ALFs also displayed contrasting electrostatic characteristics. Notably, while ALFs from Groups B, C and F showed cationic properties, Groups A, D, E and G were composed of anionic ALFs (Figure 1B). However, independently of their differences in primary structure and biochemical properties, the seven ALFs shared a similar three-dimensional architecture: three α-helices packed against a four-stranded β-sheet (Figure 1C). Members of the seven groups were identified in at least four different shrimp species (*Farfantepenaeus aztecus*, *L. vannamei*, *M. japonicus* and *P. monodon*) (Table S1). Remarkably, Group G ALFs were the only members that were not identified in the genus *Fenneropenaeus* (*F chinensis*, *F. indicus* and *F. penicillatus*). On the other hand, two different members from Group C were identified in *F. chinensis* (*Fc*ALF2: JX853775 and *Fc*ALF3: JX853776), *M. japonicus* (*Mj*ALF-C1: AB210110 and *Mj*ALF-C2: KU160498) and *P. monodon* (ALF*Pm*6: JN562340 and ALF*Pm*7: KX431031).

Our knowledge of ALF intraspecific sequence diversity was also enriched by the discovery of novel sequences in *F. aztecus* (Groups A to G), *F. penicillatus* (Groups A to F), *L. vannamei* (Groups E to G) and *P. monodon* (Groups D to G). Besides, according to our in silico analyses, some ALFs from *M. japonicus* were classified in a different Group to that previously categorized by Jiang and colleagues [11]. For instance, the sequence *Mj*ALF-A2 [11] is actually a member of Group G and not an ALF from Group A, whereas the cationic *Mj*ALF-D1 (GenBank: KU160499) belongs to Group F and not to Group D (which gathers anionic sequences only). More surprisingly, the sequence *Mj*ALF-E1 (GenBank: KY627760), previously classified as a cationic member of Group E, did not fit in any ALF Group. Indeed, its mature sequence contains an additional cysteine residue (apart of the two cysteines holding the central β-hairpin structure) that is not found in ALFs from either marine chelicerates or crustaceans. Interestingly, coding sequences related to *Mj*ALF-E1 were also found in *P. monodon* and *L. vannamei*. Unlike the three-cysteine-containing sequences from *M. japonicus* and *P. monodon*, the sequences identified in *L. vannamei* contain four cysteines (Figure S1).

### 2.2. ALF Sequence Diversity Is Gene-Encoded

To gain insights into the origin of the molecular diversity of the shrimp ALF family, we searched for ALF gene sequences in both annotated (GenBank Nucleotide) and non-annotated (Whole-Genome Shotgun Contigs) databases. From our in silico mining analysis, seven unique genomic sequences were identified in *P. monodon*: *ALFPm2* from Group A (GenBank: EF523561), *ALFPm3* from Group B (GenBank: EF523562), *ALFPm6* from Group C (GenBank: JN562340), *ALFPm8* from Group D (GenBank: NIUS010164210), *ALFPm9* from Group E (GenBank: NIUS010076396), *ALFPm10* from Group F (GenBank: NIUS011801312) and *ALFPm11* from Group G (GenBank: NIUS010749450). Each genomic sequence corresponded to a specific ALF member and we found no evidences that ALFs from different Groups could be encoded by a same genomic sequence.

Despite their differences in terms of sequence signatures, all genes shared a similar structural organization: three exons interrupted by two introns (Figure 1D). Every sequence presents a second exon that encodes the four stranded β-sheets, with the two cysteines delimiting the central β-hairpin. This structure holds the seven charged residues involved in LPS binding and is considered as the functional domain of ALFs. As shown in Figure 1D, not all *P. monodon* ALFs contain those conserved residues found in ALF*Pm*3 from Group B [6]. Regarding the other gene regions, the first exon covers the 5′-untranslated region (UTR), the leader sequence and the hydrophobic N-terminal portion of the mature peptide (the first α-helice), and the third exon encodes the two C-terminal α-helices of the mature peptide and the 3′-UTR.

### 2.3. ALFs Evolved from Gene Duplication Events before Shrimp Speciation

**Figure 2:**
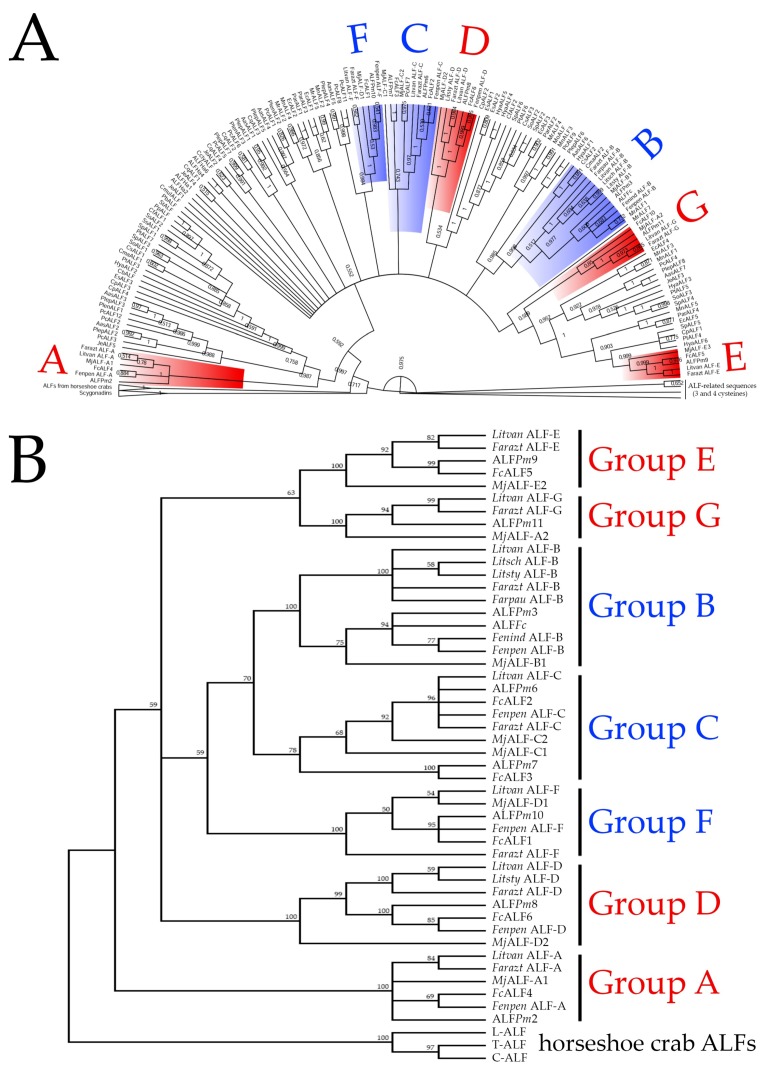
ALFs form a diverse antimicrobial peptide family in decapod crustaceans. (**A**) Bayesian and (**B**) neighbor-joining trees of ALFs from decapod crustaceans and marine chelicerates (horseshoe crabs). Cationic and anionic ALF groups from penaeid shrimp are displayed in blue and red, respectively. Posterior probabilities (Bayesian) and bootstrap values (neighbor-joining) higher than 50% are shown in the nodes. The list of the ALF sequences included in analyses (annotations, sequences and GenBank accession numbers) is provided in Table S1.

In order to unravel the phylogenetic relationships of the shrimp ALFs, phylogenetic reconstructions were performed with ALF sequences from 38 species of decapod crustaceans (suborders Dendrobranchiata and Pleocyemata) and three species of marine chelicerates (the horseshoe crabs *Carcinoscorpius rotundicauda*, *L. polyphemus* and *T. tridentatus*) (Table S1). Additionally, we analyzed the ALF-related sequences containing three and four cysteine residues and scygonadins (anionic AMPs from crabs that contain two cysteines flanking 17 amino acid residues [12]). Our Bayesian phylogenetic analysis revealed that ALFs comprise a large and diverse gene family in decapod crustaceans. The first striking piece of information is that the three/four-cysteine-containing peptides (including *Mj*ALF-E1), as well as scygonadins, are not authentic members of the ALF family since they form a separate and distant clade from all other sequences (Figure 2A). Indeed, the ALF clade gathered sequences from both crustaceans and marine chelicerates. Regarding the crustacean group, ALFs were split into two main clades (Figure 2A). The first clade included ALFs from Group A, while the second clade gathered ALFs from six additional groups (B to G). Interestingly, sequences from non-penaeid species (Pleocyemata) were found in all shrimp ALF groups, but they also formed exclusive groups distinct from those found in penaeids (Figure 2A).

Then, an additional phylogenetic tree was constructed to determine the phylogenetic relationships among the seven shrimp ALF groups (A to G) (Figure 2B). In this tree, shrimp ALFs clustered into two main clades: a first clade containing ALFs from Group A and a second clade divided into three branches. Within the second clade, all cationic shrimp ALFs (Groups B, C and F) clustered into one branch, and anionic ALFs from Groups E and G clustered into a second branch (Figure 2B). Group D ALFs clustered in a third branch (Figure 2B). Altogether, our results suggest that the sequence diversity found in the ALF family was likely driven by gene duplication events before the divergence of decapod crustaceans (Dendrobranchiata and Pleocyemata). Notably, the gene expansion and subsequent diversification of the ALF family seems to have occurred in crustaceans and not in marine chelicerates. Actually, in marine chelicerates, only one ALF type was identified (Table S1).

### 2.4. ALFs Are All Expressed in Individual Shrimps and Differentially Modulated in Response to Tissue Damage

**Figure 3:**
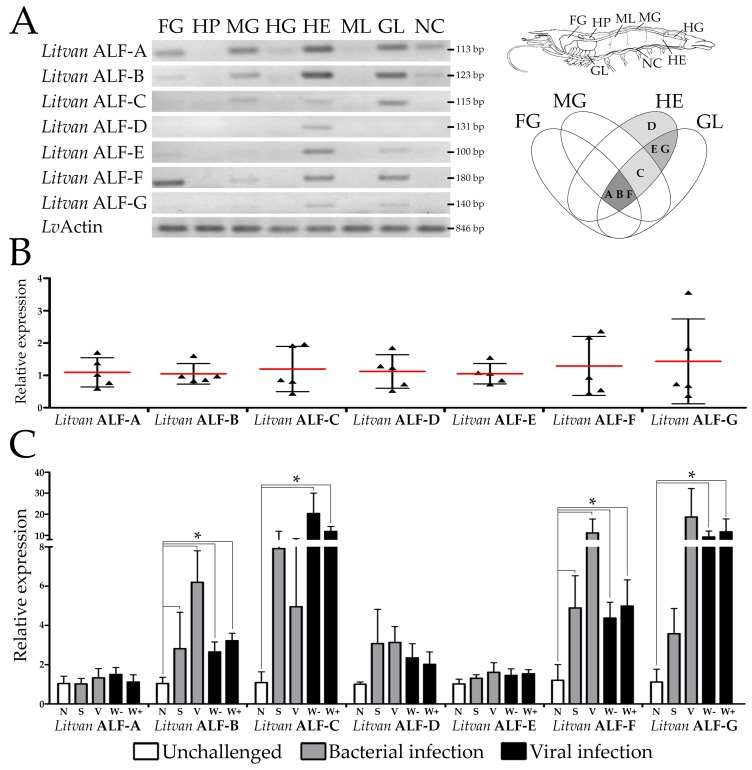
Tissue expression distribution of shrimp ALFS and gene modulation in hemocytes in response to pathogen challenge and tissue damage. (**A**) Semiquantitative RT-PCR analysis of *L. vannamei* ALFs in shrimp tissues: foregut (FG), hepatopancreas (HP), midgut (MG), hindgut (HG), hemocytes (HE), muscle (ML), gills (GL) and nerve cord (NC). The expression of the *Lv*Actin gene was used as endogenous control. The anatomic location of the tissues is indicated in the shrimp image. The Venn diagram summarizes the main sites of expression of *L. vannamei* ALFs. (**B**) mRNA basal levels of *L. vannamei* ALFs in the circulating hemocytes from five individual shrimp. (**C**) Gene expression profile of ALFs in the hemocytes of shrimp at 48 h after experimental infections with *V. harveyi* (grey bars) or WSSV (black bars). Results are presented as mean ± standard deviation of relative expressions (three biological replicates) and statistical differences are indicated by asterisks (*) (one-way ANOVA/Tukey, *p* < 0.05). N: naïve (non-stimulated) shrimp (white bars), S: sterile seawater injury control, V: *V. harveyi* ATCC 14126 (6 × 10^7^ CFU/animal), W−: tissue homogenate inoculum prepared from WSSV-free shrimp, W+: WSSV (3 × 10^2^ viral particles/animal).

We further focused on the transcriptional profiles of shrimp ALFs in terms of tissue distribution. For gene expression analyses, we considered the seven ALFs from the Pacific white shrimp *L. vannamei* (*Litvan* ALF-A to -G). First, the gene expression distribution of *Litvan* ALFs was assessed in eight different tissues of healthy juveniles by semiquantitative RT-PCR analysis. Overall, *Litvan* ALFs were mainly detected in circulating hemocytes and gills (Figure 3A). Transcripts of *Litvan* ALF-A and *Litvan* ALF-B were detected in foregut, midgut, hemocytes, gills and nerve cord, while the expression of *Litvan* ALF-C was observed in midgut, hemocytes, and gills (Figure 3A). Besides, while the expression of *Litvan* ALF-E and *Litvan* ALF-G was exclusively detected in hemocytes and gills, *Litvan* ALF-F was mainly expressed in the foregut (Figure 3A). Unlike the other ALF groups, the expression of *Litvan* ALF-D was only detected in hemocytes (Figure 3A). For all genes, no signals were observed in hepatopancreas, hindgut and muscle (Figure 3A).

ALF gene expression was then studied in response to infections and wounding. We first asked whether ALF genes were all transcribed in a single animal or whether their diversity reflected inter-individual sequence variability. Transcripts of the seven ALF genes were detected in the circulating hemocytes of every individual shrimp, as determined by RT-qPCR (Figure 3B). However, important variation was observed in the basal transcription of each gene among individuals (Figure 3B). While the basal gene expression of *Litvan* ALF-A to *Litvan* ALF-F varied from 2- to 6-fold among individuals, variations up to 11.3-fold were found for *Litvan* ALF-G gene expression (Figure 3B).

Next, we analyzed the gene expression profile of *Litvan* ALFs in response to microbial challenge and injury. Two unrelated shrimp pathogens were chosen: the Gram-negative *V. harveyi* and the White spot syndrome virus (WSSV). The transcriptional response of *Litvan* ALFs was quantified by RT-qPCR in shrimp hemocytes 48 h after infections. This time point was chosen on the basis of previous studies from our group [13,14,15]. Anionic ALFs from Groups A, D and E did not respond to pathogens nor to injury (Figure 3C). Conversely, cationic ALFs (Groups B, C and F) and the anionic Group G ALF showed significant changes in expression only in response to tissue injury. Indeed, the expression of *Litvan* ALF-B (2.6-fold), *Litvan* ALF-C (18.7-fold), *Litvan* ALF-F (3.6-fold) and *Litvan* ALF-G (8.3-fold) was significantly induced in circulating hemocytes after the injection of a tissue homogenate prepared from shrimp muscle (Figure 3C). Similarly, the expression of *Litvan* ALF-B (2.7-fold) and *Litvan* ALF-F (4.1-fold) also increased after the injection of sterile seawater. The pathogens (*V. harveyi* and WSSV) did not modulate further ALF expression. Notably, independently of the experimental condition, a high variability in gene expression was observed for all ALFs (Figure 3C).

### 2.5. Some ALF Genes Are Transcribed Early in Shrimp Development, while Others Are Mainly Expressed in Juveniles

**Figure 4:**
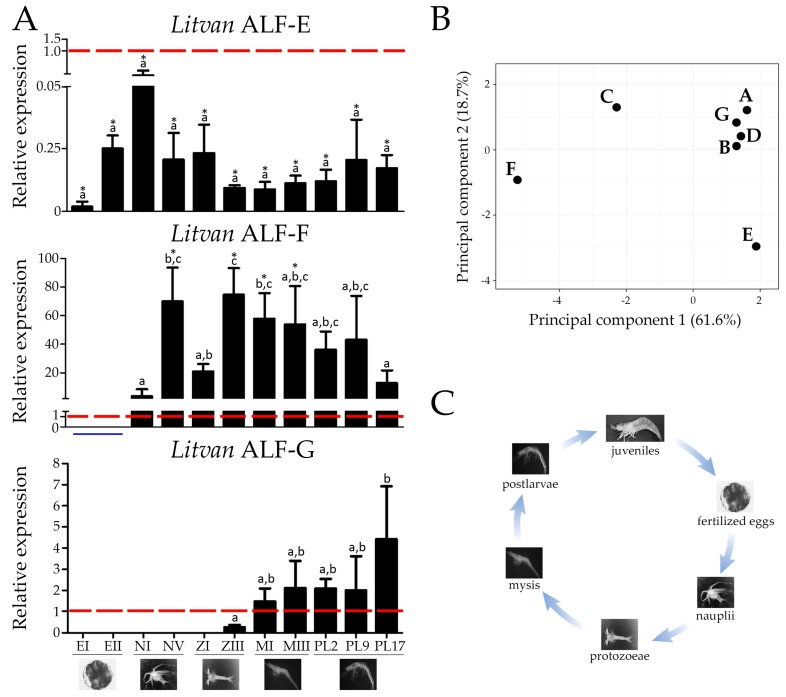
Expression of ALFs during shrimp development. (**A**) Gene expression profile of *Litvan* ALF-E, -F and -G in twelve developmental stages: fertilized eggs at 0–4 h post-spawning (EI), fertilized eggs at 7–11 h post-spawning (EII), nauplius I (NI), nauplius V (NV), protozoea I (ZI), protozoea III (ZIII), mysis I (MI), mysis III (MIII), postlarva 2 (PL2), postlarva 9 (PL9), postlarva 17 (PL17). Representative images of the developmental stages are indicated at the bottom of the graph. Results are present as mean ± standard deviation. The red dotted line indicates the expression in hemocytes from juveniles while the solid blue underline highlights the stages at which the expression was detected (valid dissociation curve) but not quantified (Cq values higher than the limit of quantification). Different letters indicate significant differences among the developmental stages and asterisks (*) shows significant differences between each developmental stage and hemocytes from juveniles (one-way ANOVA/Tukey, *p* < 0.05). (**B**) Results of principal component analysis showing the relationship among the expression profile of *L. vannamei* ALFs during shrimp development. (**C**) The life cycle of the Pacific white shrimp *L. vannamei*.

Finally, we studied the expression of the three new *L. vannamei* ALFs (Groups E, F and G) at different stages of shrimp development: fertilized eggs, nauplii, protozoeae, mysis, postlarvae and juveniles. The expression profile of *Litvan* ALFs from Groups A to D was previously reported [16]. Three distinct patterns of expression were observed for ALF groups E to G over *L. vannamei* development. ALFs from Groups E and F were detected at all developmental stages, but Group F expression could only be quantified from nauplius stages (Figure 4). Group E expression did not vary significantly over the entire shrimp development, from larvae to juveniles. In contrast, Group F expression was maximum in protozoea III (ZIII) and then decreased significantly in juveniles (PL17) (Figure 4). Finally, Group G ALF was only expressed from protozoea III (ZIII), and its expression increased significantly up to juvenile stages (PL17) (Figure 4).

### 2.6. Sequence Diversity of Shrimp ALFs Results in Distinct Antimicrobial Properties

**Figure 5:**
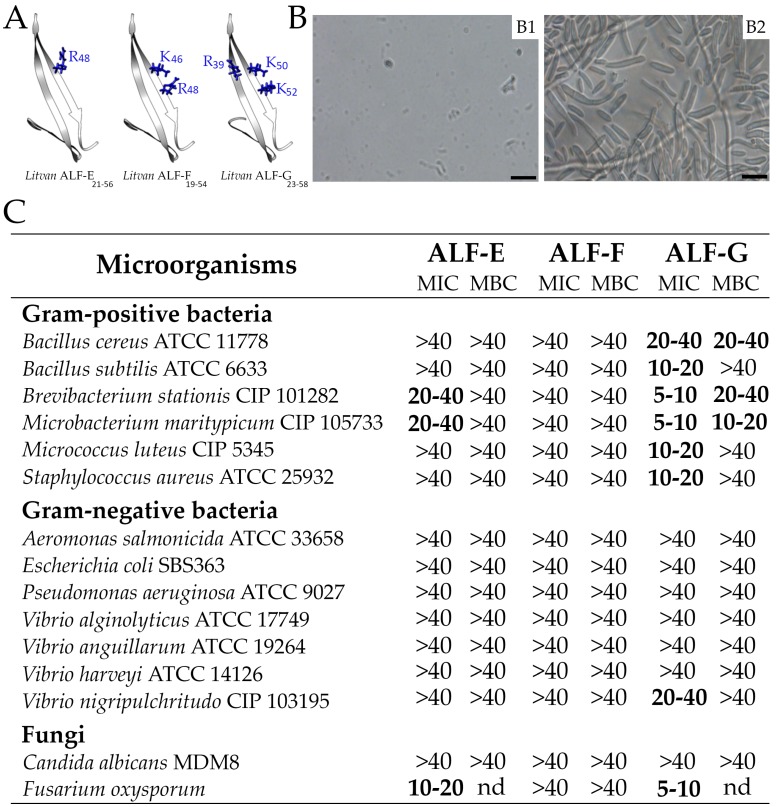
The antimicrobial spectrum of the novel shrimp ALFs. (**A**) Predicted three-dimensional structure of the central β-hairpin of *Litvan* ALF-E, -F and -G. The structural models were built based on the NMR structure of ALF*Pm*3 (PDB: 2JOB). Conserved residues involved in LPS binding of ALF*Pm*3 are displayed in blue. (**B**) Representative images of the effects of ALFs against *F. oxysporum*: (B1) fugal spore inhibition (antifungal effect); (B2) fungal spore germination (no antifungal activity). Bars = 20 µm. (**C**) Spectrum of antibacterial and antifungal activities of synthetic peptides based on the central β-hairpin of *Litvan* ALF-E, -F and -G. Minimum inhibitory (MIC) and Minimum bactericidal (MBC) concentrations are expressed in µM. nd: non-determined.

The functional domain (central β-hairpin) of the three novel ALF members identified in *L. vannamei* (Groups E, F and G) was generated by chemical synthesis to evaluate their antimicrobial properties. Indeed, this functional domain is considered a good proxy of the full-length ALF antimicrobial properties [9,10,17]. Minimal inhibitory concentration assays were performed against Gram-positive and Gram-negative bacteria and fungi (yeast and filamentous) (Figure 5). From the three synthetic peptides, *Litvan* ALF-G_34-55_ displayed the broadest range of antimicrobial activity, being effective against all tested Gram-positive bacteria, the Gram-negative *V. nigripulchritudo* and the filamentous fungus *F. oxysporum* (Figure 5). This peptide could also affect the growth of the Gram-negative bacteria *E. coli* and *V. harveyi* at 40 µM (data not shown), but total inhibition was only observed against *V. nigripulchritudo*. Additionally, *Litvan* ALF-G_34-55_ exhibited bactericidal activity against the Gram-positive bacteria *B. cereus*, *B. stationis* and *M. maritypicum*. On the other hand, synthetic β-hairpins of *Litvan* ALF-E_32-53_ could inhibit only the growth of marine Gram-positive bacteria (*B. stationis* and *M. maritypicum*) and *F. oxysporum* (Figure 5). Notably, no antimicrobial activity was observed for *Litvan* ALF-F_30-51_ β-hairpin even at 40 µM. None of the synthetic peptides was able to inhibit the growth of the Gram-negative bacteria *A. salmonicida*, *P. aeruginosa*, *V. alginolyticus* and *V. anguillarum*, and of the yeast *C. albicans*. Thus, according to their synthetic β-hairpin, Group G and to a lower extent Group E ALFs show a broad spectrum of antimicrobial activities, whereas Group F is devoid of antifungal and antibacterial activity. However, in agreement with a very poor conservation of residues involved in LPS binding (Figure 1D), ALFs from Groups E‒G were almost inactive against Gram-negative bacteria (Figure 5C).

## 3. Discussion

We showed here that shrimp ALFs are composed of seven distinct members (Groups A to G) with contrasting biochemical properties, activities and expression patterns. Particularly, ALF sequences were found to vary from cationic to anionic with important consequences on their antimicrobial activities. Overall, ALFs comprise the most diverse AMPs found in penaeid shrimp. Indeed, such diversity has not been observed in any other gene-encoded AMP families from shrimp, which are exclusively composed of cationic (penaeidins and crustins) or anionic (stylicins) members [8,12]. ALF diversity is encoded by at least seven genes that arose from successive duplications and subsequent mutations (nucleotide substitutions and insertion/deletion events) before decapod crustacean speciation occurred. This indicates that strong evolutionary pressures have driven the functional diversification of ALF genes, giving rise to neo- or sub-functionalization and retention in the shrimp genome.

We found that shrimp ALFs are paralogous genes that evolved before the speciation of the suborder Dendrobranchiata (penaeid shrimp). Indeed, ALF diversity, which is the subject of the present study, goes beyond penaeid shrimp and extends to other decapod species from the suborder Pleocyemata (including crayfish, crabs, lobsters, freshwater prawns, etc.). Some ALF members from non-penaeid decapods fall into the seven groups characterized here for penaeid shrimp. However, the ALF diversity found in the suborder Pleocyemata is different from penaeid shrimp (Dendrobranchiata). It is likely that the remarkable gene expansion and diversification of ALF sequences through gene duplication and subsequent mutation have fueled adaptation to different lifestyles and environments (and their associated pathogens) among crustaceans. Here, we have focused our study on shrimp ALFs, as a good sub-representative of ALF diversity. In order to support our hypothesis, we showed that the biological activities and expression patterns of ALF genes have diverged. In particular, we found that some ALFs are antimicrobial, whereas others are not. Some are expressed early during shrimp development, whereas others are expressed in late developmental stages. Finally, ALFs differ in their tissue distribution and responses to tissue damage. However, much more biological data are still needed on the expression and functions of the different ALF members to understand ALF evolution. This ambitious objective will require the development and use of emerging gene-silencing technologies (such as CRISPR-Cas9 and RNA interference) to achieve specific invalidation of closely-related genes in crustaceans and further phenotyping. Similarly, molecular tools such as in situ hybridization could reveal the tissue specificity of ALFs and thus, they would help in uncovering other biological functions. Finally, a classification of all crustacean ALFs (from both decapod and non-decapod species) based on robust phylogenetic reconstructions may avoid misleading classifications and lead to consensus among researchers.

With the identification of two novel ALF groups, we found that ALFs from Groups E and G share a common ancestor gene. Interestingly, Group G is lacking in species of the genus *Fenneropenaeus* whereas it is found in species of the genera *Farfantepenaeus*, *Litopenaeus*, *Marsupenaeus* and *Penaeus*. Although it cannot be ruled out that data are missing from publicly accessible databases, the absence of Group G in *Fenneropenaeus* could result from a gene loss event within this genus. Alternatively, the duplication event that originated these two genes may not have occurred in the genus *Fenneropenaeus*. Indeed, the evolutionary history of each group traced a particular trajectory in each shrimp species. For instance, while Group C ALFs from *F. chinensis* (*Fc*ALF2 and *Fc*ALF3 [17]), *M. japonicus* (*Mj*ALF-C1 and *Mj*ALF-C2 [11]) and *P. monodon* (ALF*Pm*6 and ALF*Pm*7 [18,19]) are composed of two members, in other penaeids this group appears to be composed by a single gene. However, we do not favor this last hypothesis as Group G is found in a diversity of penaeid species. Interestingly, in *L. vannamei*, Group G ALF was shown here to (i) have broader and more potent antimicrobial activity than Group E ALF, according to their β-hairpin activity, and (ii) to be expressed at late developmental stages whereas Group E expression tends to decrease over ontogenesis. Therefore, it is tempting to speculate that Group E confers antimicrobial protection at larval stages when Group G is still not expressed, whereas Group G provides a selective advantage to the *Litopenaeus* genus in facing infections at juvenile and adult stages when they are more exposed to different environmental challenges.

We showed that the expression of the seven ALF genes is simultaneous in the circulating hemocytes of a single shrimp. This result is particularly interesting because it suggests that the different ALF members may act synergistically to improve their antimicrobial properties. However, it is still unknown whether they are produced by the same hemocyte populations. Comparatively, the different penaeidin members of *L. vannamei* (*Litvan* PEN1/2, -3 and -4) are constitutively expressed by the granular cell populations [20]. Although the expression of ALFs has been detected in hemocytes from juveniles, some members (Groups C, E and F) appeared to be transcribed in larval stages of shrimp development that precede the emergence of these immune cells [21]. Instead, the expression of ALFs from Groups A, B, D and G was quite similar to that observed for other shrimp AMPs that are exclusively produced by hemocytes [16]. On the one hand, the expression of ALFs early in development could be the result of maternal transmission [16,22] but, on the other hand, those transcripts might originate from other shrimp tissues. Interestingly, in different species, including *L. vannamei*, the expression of ALFs from Groups C and F was mainly detected in other tissues (digestive system, gills, eyestalk) than in the circulating hemocytes [17,18,19]. However, only the expression of ALFs from Group B (ALF*Pm*3 from *P. monodon*) was studied by immune staining [23]. More knowledge about the precise sites of ALF production will contribute to understand the involvement of these AMPs in shrimp epithelial defenses, especially those occurring in gills and intestines [15].

One important finding from this study concerns the differential gene expression pattern of shrimp ALFs in response to various challenges. Indeed, the different ALF genes found across penaeids showed to be responsive to various shrimp pathogens, from viruses to bacteria and filamentous fungi [11,14,15,18]. Moreover, RNA interference (RNAi)-mediated gene silencing assays have confirmed that ALFs are directly involved in shrimp survival to infectious diseases [18,24,25]. Additionally, our results provided new evidences for the role of ALFs in other biological processes. Interestingly, while ALFs from Groups A, D and E were not regulated, the expression of the other ALF genes was induced in response to tissue damage. Particularly, ALFs from Groups C and G were shown to be responsive to a tissue homogenate prepared from shrimp muscle (injury control for the WSSV infection), suggesting that they can be modulated by danger/damage-associated molecular patterns (DAMPs). This nonspecific transcriptional response could be associated with additional biological roles involving the promotion of wound healing and the rapid regeneration of tissues [20]. Additionally, we showed that some ALFs are modulated in the shrimp gut in response to infections, suggesting that ALFs can act as a first line of defense in tissues continuously exposed to microbe-rich environments [15,19]. Therefore, it is possible that those ALF variants have evolved novel functions associated with the control of the intestinal microbiota. The shrimp intestinal microbiota is a complex and dynamic community that is directly influenced by both biotic and abiotic factors [13], but probably it is also by the constitutive expression of immune-related genes. In fact, RNAi experiments revealed that ALFs from Groups B [18] and C [25] play an essential role in the control of the bacterial communities residing in the hemolymph. However, more functional genomic studies are required to understand the role of ALF in shrimp intestinal defenses.

Another relevant conclusion taken is that the antimicrobial activity of the functional domain of ALFs (central β-hairpin) is associated with its primary sequence rather than to its charge. Despite their differences in primary structure and biochemical features, the seven ALF groups shared a similar tertiary structure. However, the residues involved in LPS binding are not conserved among the seven groups, confirming the neo-functionalization hypothesis proposed by Rosa and colleagues [10]. Indeed, LPS binding has been demonstrated for the limulus ALF sequence, which shares a common ancestor with all shrimp ALFs. Taking into account previous studies [9,10,11,17] and the present results, shrimp ALFs have proved to display a diverse spectrum of antimicrobial activity. While some members exhibited a broad range of antimicrobial activity (Groups B and G), some others displayed limited (Groups A, C and E) or very weak action (Groups D and F). One possible explanation is that the effectiveness of the antimicrobial activity of each ALF group is directly proportional to the amount of positively charged amino acids in its central β-hairpin structure [26]. However, we showed that the highly cationic central β-hairpin structure of *Litvan* ALF-F_19-54_ (p*I* = 9.24) was not active against the microorganisms tested in this study. Likewise, synthetic β-hairpins of the *Fc*ALF1 (Group F) from *F. chinensis* was also poorly active against both Gram-positive and Gram-negative bacteria [17]. Thus, besides their overall net charge, other features may interfere directly on their biological activities. Given these results, the determination of the amino acid residues involved in the interaction with other microbial surface molecules (peptidoglycan, lipoteichoic acid, β-glucans, etc.) may provide valuable information of the mechanism of action of ALFs against other microorganisms beyond Gram-negative bacteria [7].

## 4. Materials and Methods

### 4.1. Database Searches and Phylogenetic Reconstructions

ALF sequences were methodically collected from publicly accessible databases and used for the search of homologous sequences in both annotated and non-annotated databases. Only full-length coding sequences were considered. Homology searches were performed using tBLASTx at NCBI. Exon-intron boundaries were defined by alignment of the cDNA and genomic sequences. All nucleotide sequences were manually inspected and analyzed using open-access bioinformatics tools. Three-dimensional models for *L. vannamei* ALFs were built with SWISS-MODEL (https://swissmodel.expasy.org/) using ALF*Pm*3 NMR resolution (PDB: 2JOB1) as a template. Deduced amino acid sequences were aligned using MAFFT multiple alignment program (https://mafft.cbrc.jp/alignment/server/). Bayesian phylogenetic analysis was conducted in MrBayes 3.1.2 (http://mrbayes.sourceforge.net/), using WAG + G as substitution model, with two runs of 10^7^ generations, sample rate of 1000 and burn-in of 25%. Neighbor-joining analysis was conducted in MEGA X [27]. Bootstrap sampling was reiterated 1000 times using a 50% bootstrap cutoff. Trees were drawn using FigTree v1.4.2 (http://tree.bio.ed.ac.uk/software/figtree/).

### 4.2. Animals and Tissue Collection

*Litopenaeus vannamei* juveniles (10 ± 2 g) and at different development stages were obtained from the Laboratory of Marine Shrimp (Federal University of Santa Catarina, Florianópolis, Brazil). Each developmental stage was identified microscopically and collected as previously described [16] while juveniles were acclimated in controlled conditions for at least one week before any experimentation. Hemolymph was collected from the ventral sinus into a precooled modified Alsever solution (27 mM sodium citrate, 336 mM NaCl, 115 mM glucose, 9 mM EDTA, pH 7.0) and hemocytes were isolated by centrifugation. After hemolymph collection, the following tissues were harvested by dissection: foregut, hepatopancreas, midgut, hindgut, muscle, gills and nerve cord. Tissues were rinsed in Tris-saline solution (10 mM Tris, 330 mM NaCl, pH 7.4), homogenized in TRIzol reagent (Thermo Scientific, Asheville, NC, USA) and processed for semiquantitative RT-PCR analysis.

### 4.3. Experimental Infections

Two unrelated shrimp pathogens were chosen for experimental infections, the Gram-negative *Vibrio harveyi* and the White spot syndrome virus (WSSV). For the bacterial infection, 6 × 10^7^ CFU/animal of *V. harveyi* ATCC 14126 under 100 µL sterile seawater (SSW) or 100 µL SSW (injury control) were injected. For the viral infection, shrimp were injected with 100 µL of a WSSV inoculum containing 3 × 10^2^ viral particles. The WSSV inoculum was prepared from muscle tissues of WSSV-infected shrimp as previously described [14]. Animals injected with 100 µL of a tissue homogenate prepared from WSSV-free shrimp were used as injury control for the viral infection. At 48 h post-infections, hemocytes were collected, pooled (three pools of five animals per condition) and processed for gene expression analysis. Naïve (non-stimulated) animals were used as a control for all experimental conditions.

### 4.4. Semiquantitative RT-PCR Analysis for Tissue Distribution of Gene Expression

Total RNA was extracted using TRIzol reagent (Thermo Scientific, Asheville, NC, USA) according to the manufacturer’s protocol. RNA samples were treated with DNase I (Thermo Scientific) at 37 °C for 15 min and precipitated with 0.3 M sodium acetate (pH 5.2) and isopropanol (1:1; *v*:*v*). RNA amount and quality were assessed by spectrophotometric analysis and the integrity of total RNA was analyzed by 0.8% agarose gel electrophoresis. First strand cDNA was synthesized from 1 µg of total RNA using the RevertAid Reverse Transcription kit (Thermo Scientific, Asheville, NC, USA) and oligo(dT)_12-18_ primers. PCR reactions were carried out in a 15-µL reaction volume containing 1 µL cDNA, 2 mM MgCl_2_, 0.4 mM dNTP Mix, 0.4 µM of each primer (Table 1) and 1 U Taq DNA Polymerase (Sinapse, São Paulo, SP, Brazil). PCR conditions were as follows: 1 cycle of denaturation at 95 °C for 10 min followed by 30–35 cycles of 95 °C for 30 s, 60 °C for 30 s and 72 °C for 30 s. PCR products were analyzed by electrophoresis (1.5% agarose gel) and stained by ethidium bromide. The expression of the *Lv*Actin gene was used as endogenous control.

### 4.5. Fluorescence-Based Reverse Transcription Real-Time Quantitative PCR (RT-qPCR)

RT-qPCR amplifications were performed in a final volume of 15 μL containing 0.3 μM of each primer (Table 1), 7.5 μL of reaction mix (Maxima SYBR Green/ROX qPCR Master Mix 2×; Thermo Scientific, Asheville, NC, USA) and 1 μL of cDNA. The RT-qPCR program was 95 °C for 10 min, followed by 40 cycles of 95 °C for 15 s and 60 °C for 1 min. Melt curve analysis was performed to evaluate primer specificity. The eukaryotic translation elongation factor 1-alpha (*Lv*EF1α) and the ribosomal protein *Lv*L40 were used as reference genes of expression data in hemocytes. Relative transcript levels were determined by the comparative standard curve method using a standard curve derived from 2-fold dilution series of a cDNA pool of all samples. Differences we considered significant at *p* < 0.05 (one-way ANOVA and Tukey’s multiple comparison test). Gene expression of ALFs during shrimp development was assessed in twelve developmental stages as previously described [16].

### 4.6. Peptide Synthesis, Oxidation and Characterization

Synthetic peptides based on the central β-hairpin of ALF-E (*Litvan* ALF-E_32-53_: GCYVNRSPYLKKFEVHYRADVKCG), ALF-F (*Litvan* ALF-F_30-51_: GCTYFVTPKVKSFELYFKGRMTCG) and ALF-G (*Litvan* ALF-G_34-55_: GCSYSTRPYFLRWRLKFKSKVWCG) were obtained in a Liberty Blue automated microwave peptide synthesizer (CEM Corp, Matthews, NC, USA) using Fmoc-protected amino acids (Iris Biotech GmBH (Marktredwitz, Germany) and Rink Amide AM resin (loading: 0.6 meq/g). Fmoc deprotection was carried out with 20% *v*/*v* piperidine in DMF, couplings were performed with DIC/OxymaPure activation (1/1 eq) and additional couplings with TBTU/DIEA/OxymaPure activation (1/2/1 eq). Peptides were cleaved with TFA/TIS/DOT/H_2_0 (92.5/2.5/2.5/2.5) [trifluoroacetic acid/triisopropylsilane/2,2-(ethylenedioxy)-diethanethiol/ultrapure water] and purified by RP-HPLC (JASCO Corp., Tokyo, Japan) on a XBridge™ BEH C18 column (100 × 4.6 mm, 3.5 µm) (Waters Corp., Milford, MA, USA) with a 0–70% acetonitrile-water mixture gradient over 30 min at a flow rate of 1 mL/min. Peptides were further lyophilized and analyzed by MALDI-TOF mass spectrometry in a LCMS-2020 ESI-MS (Shimadzu Corp., Kyoto, Japan) to confirm their molecular masses.

Then, peptides were oxidized as previously reported [28]. In brief, 5 mg of the crude peptide were first reduced with 10% β-mercaptoethanol (95 °C for 5 min) then dissolved in 50% (*v*/*v*) AcOH/H_2_O and later diluted in 32 mL of oxidation buffer (2 mM guanidinium chloride, 10% isopropyl alcohol and 10% dimethyl sulfoxide). The pH was adjusted to 5.8 with ammonium hydroxide. The peptide solution was subjected to air oxidation at room temperature for 18 h. The peptide solution was then acidified to pH 2.5 and purified using a SPE C18 (Waters Corp., Milford, MA, USA). The peptides were eluted with 5%, 20%, 40%, 60% and 80% acetonitrile in 0.05% TFA ultrapure water at a flow rate of 1 mL/min. The fractions were collected, and the acetonitrile was evaporated on a Savant SPD 1010 SpeedVac Concentrator (Thermo Scientific, Asheville, NC, USA). The fractions were analyzed by MALDI-TOF mass spectrometry.

### 4.7. Antibacterial and Antifungal Assays

The antimicrobial activity of synthetic peptides was assayed against the Gram-positive bacteria *Bacillus cereus* ATCC 11778, *Bacillus subtilis* ATCC 6633, *Brevibacterium stationis* CIP 101282, *Microbacterium maritypicum* CIP 105733, *Micrococcus luteus* CIP 5345 and *Staphylococcus aureus* ATCC 25932, the Gram-negative bacteria *Aeromonas salmonicida* ATCC 33658, *Escherichia coli* SBS363, *Pseudomonas aeruginosa* ATCC 9027, *Vibrio alginolyticus* ATCC 17749, *Vibrio anguillarum* ATCC 19264, *Vibrio harveyi* ATCC 14126 and *Vibrio nigripulchritudo* CIP103195, the yeast *Candida albicans* MDM8 and the filamentous fungus *Fusarium oxysporum*.

Minimum inhibitory concentrations (MICs) were determined in duplicate by the liquid growth inhibition assay, as previously described [29]. MIC values are expressed as the lowest concentration tested that causes 100% growth inhibition. Poor Broth (PB: 1% peptone, 1% NaCl, pH 7.2) was used for standard bacteria, while PB supplemented with 0.5 M NaCl (PB–NaCl) was used as a culture medium for *Vibrio* strains. For *B. stationis* and *M. maritypicum* cultures, PB–NaCl medium was supplemented with 20 mM KCl, 5 mM MgSO_4_ and 1.5 mM CaCl_2_. Potato dextrose broth (Kasvi, São José dos Pinhais, PR, Brazil) at half strength was used for cultures of *F. oxysporum* while Sabouraud medium (1% peptone, 4% glucose, pH 5.6) was used for yeast cultures. The growth of bacteria and yeast was monitored spectrophotometrically (λ = 595 nm), while *F. oxysporum* hyphae formation was observed in an inverted microscope. After MIC determination, bacterial cultures were plated in nutrient agar for 24–48 h for the determination of the bactericidal activity of the synthetic peptides.

## 5. Conclusions

In conclusion, the combination of our molecular, transcriptional and functional data revealed that ALFs comprise the most diverse AMP family found in penaeid shrimp. We showed that they are composed of seven members encoded by different genes that follow a diverse pattern of expression. Our results also strongly suggest that the expansion and diversification of shrimp ALFs have shaped novel functions for this AMP family beyond their primary antibacterial properties. Thus, ALFs represent an attractive model to explore the impacts of the molecular diversity of immune-related genes on host‒microbe interactions. Finally, ALFs possess the broadest spectrum of antimicrobial activity when compared to other shrimp AMPs. These bioactive peptides undoubtedly show biotechnological potential for the development of novel antibiotics derived from AMPs, as well as for the development of selective breeding programs.

## Figures and Tables

**Table 1:** Nucleotide sequences of primers used in this study.

Gene	Forward Primer (5′-3′)	Reverse Primer (5′-3′)	Amplicon
*Lv*Actin ^1^	TAATCCACATCTGCTGGAAGGTGG	TCACCAACTGGGATGACATGG	846 bp
*Lv*Actin ^2^	CCACGAGACCACCTACAAC	AGCGAGGGCAGTGATTTC	142 bp
*Lv*EF1α ^2^	TGGCTGTGAACAAGATGGACA	TTGTAGCCCACCTTCTTGACG	103 bp
*Lv*L40 ^2^	GAGAATGTGAAGGCCAAGATC	TCAGAGAGAGTGCGACCATC	104 bp
*Lv*RpS6 ^2^	AGCAGATACCCTTGGTGAAG	GATGCAACCACGGACTGAC	193 bp
*Litvan* ALF-A ^1,2^	CTGATTGCTCTTGTGCCACG	TGACCCATGAACTCCACCTC	113 bp
*Litvan* ALF-B ^1,2^	GTGTCTCCGTGTTGACAAGC	ACAGCCCAACGATCTTGCTG	123 bp
*Litvan* ALF-C ^1,2^	ATGCGAGTGTCTGTCCTCAG	TGAGTTTGTTCGCGATGGCC	115 bp
*Litvan* ALF-D ^1,2^	TGTGTTGGTTGTGGCACTGG	CAACGAGGTCAATGTCACCG	131 bp
*Litvan* ALF-E ^1,2^	TGCTACGTGAATCGCAGTCC	CGCTTCCTCTTCCGACAATG	100 bp
*Litvan* ALF-F ^1,2^	AAGCTCTCATTCCTGGTCGG	GGGTGTAACGAAGTACGTGC	180 bp
*Litvan* ALF-G ^1,2^	CCGCTGCATGTCAAGTATCC	TCAGCAGTAGCAGTGTCAGC	140 bp

^1^ Primers for semiquantitative analysis of gene expression (RT-PCR); ^2^ Primers for quantitative analysis of gene expression (RT-qPCR).

## Supplementary Materials

The following are available online at http://www.mdpi.com/1660-3397/16/10/381/s1. Table S1: Sequences and biochemical properties of ALFs from decapod crustaceans and marine chelicerates. Figure S1: Amino acid sequence alignments of ALF-related sequences containing three and four cysteine residues. The predicted signal peptides are in bold and underlined. Asterisks (*) mark the identical amino acid residues while the cysteines are highlighted with a black background. GenBank accession numbers are indicated in brackets.
