# Supplementary figures and images for: Massive Gene Expansion and Sequence Diversification Is Associated with Diverse Tissue Distribution, Regulation and Antimicrobial Properties of Anti-Lipopolysaccharide Factors in Shrimp
Source: Mar Drugs. 2018 Oct 11;16(10):381. doi: 10.3390/md16100381 (PMC6213531; doi:10.3390/md16100381)

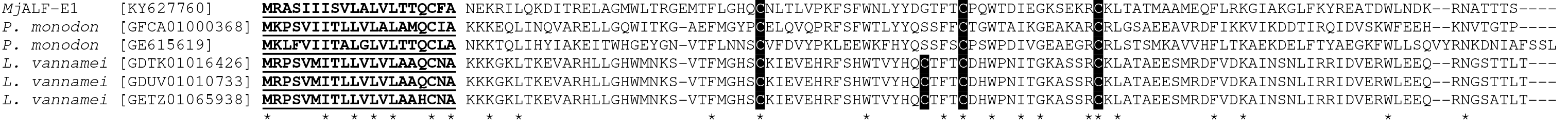

Supplement: Supplementary file 1 [file marinedrugs-16-00381-s001.zip › Figure S1 - ALF related sequences.tif]
